# Minimally Invasive Urological Procedures and Related Technological Developments—Series 2

**DOI:** 10.3390/jcm12082879

**Published:** 2023-04-14

**Authors:** Bhaskar Somani

**Affiliations:** Department of Urology, University Hospital Southampton NHS Foundation Trust, Southampton SO166YD, UK; bhaskarsomani@yahoo.com

The world of minimally invasive urology has experienced enormous growth in recent decades with technological innovations related to new techniques and equipment, better training, and the clinical adoption of translational research. There has been a substantial increase in studies related to the application of lasers both for the treatment of stones and enlarged prostates. This Special Issue in the *Journal of Clinical Medicine* (JCM) is dedicated to a collection of eleven high-quality scientific contributions. 

The first paper looked at a systematic review and meta-analysis of randomised trials on the use of coated versus non-coated urethral catheters for the prevention of catheter-associated urinary tract infections (CAUTI) [1]. A meta-analysis of 12 studies and 36,783 patients showed no significant difference in patients with long-term catheterization, and this benefit of coated catheters should be balanced against the cost to healthcare. The second paper compares the mineral content of tap water in UK and whether this was relevant to kidney stone disease (KSD) [2]. Data from 66 UK cities showed a significant variation, and depending on where someone lived, drinking 2–3 L of tap water could contribute over one-third of the recommended daily calcium and magnesium, with possible implications for KSD incidence and recurrence. 

Lasers have evolved with the advent of high-power holmium lasers, thulium fiber laser (TFL), and pulse modulation such as Moses technology [3,4,5]. There are four papers involving the use of lasers in endourology [6,7,8,9,10]. Laser efficiency and safety were compared between Holmium YAG and TFL lasers, with the latter showing higher efficiency [6]. Laser lithotripsy during ureteroscopy and stone fragmentation with comparative outcomes between ≤10 years and ≥80 years were examined by the second paper [7]. The results showed that while the former group had a higher incidence of repeated procedure, there was no difference in the overall stone-free rate (SFR) and complications between the groups. The third paper looks at low- vs. high-power lasers during the Holmium laser enucleation of the prostate (HoLEP) procedure [8]. Current evidence shows no difference in outcomes between the two and that low-power HoLEP is safe, feasible, and effective. The last paper looked at a comparison of the safety and efficacy of laser vs. pneumatic intracorporeal lithotripsy for the treatment of bladder stones in children [9]. In this prospective randomised study of 64 children, while the stone treatment was similar between groups, pneumatic lithotripsy was associated with a significantly greater risk of having at least 1 adverse effect.

Fluoroless endourology has been on the rise to minimise radiation doses. The total X-ray-free ultrasound-guided mini-PCNL in a Galdakao-modified supine Valdivia position was reported in the next paper from Taiwan on 150 patients [10]. The outcomes show that complete X-ray procedures are feasible, safe, and effective. Intrarenal pressure monitoring is also postulated to help minimise complications, but so far, there was a lack of pressure monitoring devices [11,12]. Measuring and minimising pressure would lower the risk of sepsis and other related complications [13]. Real-time intrarenal pressure control during flexible ureteorenoscopy was demonstrated in the next study using a vascular wire [14]. In this pilot study, a pressure wire was placed in the renal cavities to measure the intrapelvic pressure (IPP), with results showing the feasibility of this technique and the monitoring of IPP to identify and avoid high IPP, thereby avoiding complications. 

In the world of smart apps [15], there is a rise in wearable device technology with an annual growth rate of nearly 26% projected for India. A cross-sectional web-based survey of 495 responders exhibited a significant correlation with the adoption and acceptance of wearable devices for healthcare management in the Indian context [16]. Minimally invasive surgical therapies (MISTs) for benign prostate enlargement (BPE) have experienced a revolution with new technologies that are now on the market [17,18,19]. However, the transurethral resection of prostate (TURP) is still practiced as the primary modality of treatments in many centres. In the context of cardiovascular morbidity, patients often have to remain with the choice of preoperative antithrombotic therapy. A single-centre study from China examined venous thromboembolism (VTE) and bleeding after TURP in patients with preoperative antithrombotic therapy [20]. In a cohort of 31 patients, the authors conclude that the short-term preoperative discontinuation of therapy may help patients obtain a relatively safe opportunity for TURP surgery. This must be balanced against the risks of VTE, perioperative bleeding, and serious cardiovascular and cerebrovascular complications.

Artificial intelligence (AI) is now used in various urological conditions, including urolithiasis, benign prostate hyperplasia (BPH), and uro-oncology [21]. Similarly, radiomics is increasingly applied to the diagnosis, management, and outcome prediction of various urological conditions [22]. In a systemic review of the role of radiomics in urolithiasis, seven studies were included [23], with radiomics used to identify calculi, differentiate phleboliths, and classify stone types and compositions pre-operatively. It has also been utilized to predict outcomes and complications after endourological procedures and, hence, has great future potential. 

This Special Issue has several interesting papers that will help clinicians in decision making and treatment choices. While there is a spectrum of papers from tap water to catheters, lasers and its use in BPH and stone surgery, wearable devices, and the role of radiomics, perhaps more needs to be performed to address other aspects of a patient’s journey, such as cost and the quality of life in the management of these patients [24,25]. I am thankful to the reviewers for their professional comments and to the *JCM* team for their ongoing support with this Special Issue. Lastly, I want to thank all authors for their valuable contributions. 
